# Healthcare provider recognition of pregnancy related risks and management considerations in patients with tuberous sclerosis complex

**DOI:** 10.1186/s13023-023-03015-7

**Published:** 2024-01-02

**Authors:** Meredith Rose, David Ritter, Nishant Gupta, Leandra Tolusso, Paul Horn, Emily Wakefield, Jennifer Glass

**Affiliations:** 1 Division of Human Genetics, Cincinnati Children’s Hospital Medical Center, Cincinnati, USA; 2 Division of Neurology, Cincinnati Children’s Hospital Medical Center, Cincinnati, USA; 3 Department of Pediatrics, College of Medicine, University of Cincinnati, Cincinnati, USA; 4 Division of Pulmonary, Critical Care and Sleep Medicine, University of Cincinnati, Cincinnati, USA; 5 Medical Service, Veterans Affairs Medical Center, Cincinnati, OH USA; 6 Cincinnati Children’s Fetal Care Center, Cincinnati, USA; 7 Medical Genetics Clinic, Mary Bridge Children’s/MultiCare Health System, Tacoma, USA

**Keywords:** Tuberous sclerosis complex (TSC), Maternal pregnancy complications, Prenatal care, Screening and management, Provider risk recognition, Lymphangioleiomyomatosis (LAM)

## Abstract

**Background:**

Patients with tuberous sclerosis complex (TSC) face an increased risk of maternal health complications and worsening disease manifestations during pregnancy. There are no established consensus guidelines that address the management of pregnancy in patients with TSC and healthcare providers rely on their individual experiences and preferences to derive treatment decisions. We sought to obtain provider opinion of pregnancy related maternal complications in patients with TSC, and the common evaluation and management strategies used to address these issues.

**Methods:**

We conducted a cross-sectional survey of healthcare providers with diverse areas of expertise related to the multisystem nature of involvement in TSC. Descriptive analyses were used to analyze our three primary variables: (1) provider recognition of maternal risks/complications; (2) provider recommendations before and during pregnancy; and (3) provider/clinic protocols.

**Results:**

We received responses from 87 providers from 11 countries, with 40.7% (n = 35) seeing > 30 TSC patients yearly. The majority of providers (n = 70, 88.6%) deemed that a patient with TSC needed expert care beyond the standard of care for a typical pregnancy, with over 25% of providers reporting that they have seen lymphangioleiomyomatosis (LAM) exacerbation, seizures, and preterm labor in pregnant patients with TSC. Providers who managed patients treated with mTOR inhibitors (mTORi) also agreed that mTORi use should be stopped prior to pregnancy (n = 45, 68.2%) but there was uncertainty about when to stop the mTORi (one month 28.9%, two months 11.1%, three months 42.2%, and 6–12 months 2.2%). Additionally, there were mixed opinions on restarting mTORi in response to disease progression during pregnancy. When asked about provider or clinic specific protocols, 71.6% (n = 53) of providers stated that they do not have a clear protocol for management decisions for patients with TSC before or during pregnancy.

**Conclusion:**

Healthcare providers recognize that patients with TSC are at an increased risk for maternal health complications during pregnancy. However, there are wide inter-individual variances in practice, especially pertaining to decisions regarding mTORi use. There is a critical need to better understand the implications of pregnancy for patients with TSC, and to draft consensus recommendations to guide management decisions.

**Supplementary Information:**

The online version contains supplementary material available at 10.1186/s13023-023-03015-7.

## Background

Tuberous sclerosis complex (TSC) is a genetic condition that occurs in approximately 1 in 6,000 individuals and is caused primarily by loss-of-function variants in either the *TSC1* or *TSC2* gene, leading to overactivation of the mechanistic target of rapamycin (mTOR) pathway [1]. Symptoms develop from abnormal cell growth and proliferation in numerous organ systems including brain, skin, kidney, lung, heart, liver, and pancreas [1]. Clinical trials demonstrating the safety and efficacy of mTOR inhibitors (mTORi) to treat various manifestations of TSC have fundamentally changed the landscape of TSC management [2, 3]. mTORi have been approved by the United States Food and Drug Administration (FDA) to treat subependymal giant cell astrocytomas (SEGA), renal angiomyolipomas (AML), lymphangioleiomyomatosis (LAM), seizures, and facial angiofibromas [2, 4].

When a parent is known to have TSC, genetic counseling and fetal screening during pregnancy is recommended, as there is a 50% chance the fetus will inherit the disorder [5, 6]. It has been suggested that fetal management of at risk pregnancies should include both fetal ultrasounds and fetal echocardiograms to look for cardiac rhabdomyomas beginning at 20 weeks’ gestation, and fetal magnetic resonance imaging (MRI) to look for cerebral lesions [6–8]. While cardiac rhabdomyomas typically regress in the postnatal period without clinical consequence, they can lead to fetal dysrhythmias or blood flow obstruction through the heart resulting in the development of hydrops fetalis [7, 9]. However, despite the known risk of fetal TSC, there is a lack of clear recommendations regarding appropriate screening during pregnancy.

Case reports of mTORi use in the prenatal and immediate postnatal period in patients with TSC have been growing steadily with encouraging results [10–15]. Yet, it is unclear how often patients with TSC are treated with mTORi during pregnancy and what the fetal risk is when used in pregnant patients with TSC. mTORi are classified under category C by the FDA, meaning there is an unknown fetal teratogenicity [16]. Uncomplicated pregnancies of patients who were on mTORi for sporadic LAM have also been reported [17, 18].

While many studies have examined the fetal manifestations of TSC, few have studied the impact of pregnancy on maternal health. There have been well described maternal complications related to renal and pulmonary manifestations of TSC, including increased risk of renal AML rupture, spontaneous pneumothorax, and accelerated disease progression in patients with LAM [19–22]. There is scant literature regarding pregnancy related complications beyond renal AML and LAM. A 2005 review of 23 pregnancies of 17 patients with TSC found a 43% perinatal complication rate compared to the general population perinatal complication rate of 16% [6, 23]. These complications included both worsening of TSC related conditions (hemorrhage from ruptured renal AMLs, acute renal failure) and pregnancy specific complications (polyhydramnios, oligohydramnios, intrauterine growth restriction, preterm delivery, preeclampsia, placental abruption, and perinatal demise) [6]. In addition, children with TSC who were born to TSC-affected females were found to have more perinatal and neonatal complications when compared to children with TSC born to non-affected females [24].

Aside from counseling regarding the risk of complications associated with LAM, there is a relative paucity of advice about appropriate screening and management for patients with TSC contemplating pregnancy [5, 25]. Given that over half (60%) of the reproductive-aged adults with TSC are considering having children, with nearly 53% desiring traditional conception, there is a critical need to establish consensus-based recommendations to guide providers on proper screening and management options for patients with TSC [26].

To begin addressing this need, we conducted a survey of TSC providers across the world to better understand healthcare provider perceptions regarding the implications of pregnancy for patients with TSC and common management strategies employed in these situations by various providers. By elucidating the practice patterns across providers as well as the care gaps, we seek to aid proper management of TSC patients during pregnancy and lay the foundation to develop consensus guidelines.

## Methods

### Survey development

We designed a cross-sectional survey that was administered online in an anonymous manner to healthcare providers by using the Research Electronic Data Capture (REDCap) tools hosted at Cincinnati Children’s Hospital Medical Center. All providers were asked a uniform set of 9 demographic and 13 clinical questions; additional questions were asked to some providers based on their self-identified specialty/area of practice (Appendix 1). The survey was verbally announced and a quick response (QR) code was made available at the 2022 TSC World Conference and the 2022 International LAM Research Conference. Additionally, a link to the survey was emailed to healthcare providers through the TSC Alliance provider email list, the LAM Foundation Clinic Directors email list, and through word of mouth with maternal–fetal medicine (MFM) providers.

Survey questions were created and designed to target three primary variables: 1) Provider recognition of maternal risks/complications: this variable included provider recognition of maternal risks/complications, provider perception of the degree of risk during pregnancy, and the sources that informed the provider perceptions. We also asked about the maternal and fetal complications that providers had observed in their practice. 2) Provider recommendations before and during pregnancy: this variable captured provider recommendations to patients before and during pregnancy, including maternal and fetal evaluations/screenings. We aimed to describe the TSC-related manifestations that influence provider recommendations regarding additional evaluations/screenings and changes in mTORi use. 3) Provider/clinic protocols: this variable aimed to determine if the providers or their clinic had developed their own protocols to care for patients with TSC who either desire pregnancy or are currently pregnant.

### Statistical analysis

Descriptive analysis was completed using SAS® version 9.4 (SAS Institute Inc., Cary, NC), procedures PROC FREQ and PROC MEANS. Categorical variables were reported as proportions. “Select all that apply” responses were reported as response stratifications to describe the highest frequency responses. Confidence intervals (CI) at 95% are provided. All survey answers were used in our analysis and if a question was not answered by a respondent, that data point was dropped.

## Results

We received responses from 87 providers across 11 countries including the United States (n = 72; 82.8%), Canada (n = 3, 3.45%), Italy (n = 2, 2.30%), Brazil (n = 2, 2.30%), Japan (n = 2, 2.30%), India (n = 1, 1.15%), Spain (n = 1, 1.15%), Switzerland (n = 1, 1.15%), China (n = 1, 1.15%), South Korea (n = 1, 1.15%), and Israel (n = 1, 1.15%) (Table 1). Of the 87 responses, 73 (83.9%) were completed entirely while 14 (16.1%) were partial responses. Pulmonologists (n = 34) and neurologists (n = 28) were the most frequent provider specialties representing 72.1% of all providers. Eight (9.30%) providers were obstetrics and gynecology (OBGYN) or MFM specialists. Thirty (34.9%) providers reported seeing patients for fetal and/or pregnancy care. Thirty-five (40.7%) providers see > 30 patients a year with TSC and over 60% see young adults (n = 55) and adults (n = 58) as part of their practice. Almost half of the providers (n = 42; 48.9%) have been in practice for at least 16 years and 53 providers (61.6%) practice in a TSC clinic.

**Table 1:** Respondent demographics. *demographics were only available for 86 respondents

Respondent demographics	N = 86*
Provider specialty area (may select more than one)	
Pulmonologist	34 (39.5%)
Neurologist	28 (32.6%)
MFM	7 (8.10%)
OBGYN	1 (1.20%)
Genetics/GENETIC COUNSELING	7 (8.10%)
Nephrology	2 (2.30%)
Nursing	3 (3.50%)
Oncology	2 (2.30%)
Psychology/psychiatry	3 (3.50%)
Other (cardiology)	2 (2.30%)
Number of TSC patients seen yearly (select one)	
Never seen a patient with TSC	2 (2.30%)
0–1	9 (10.5%)
2–10	27 (31.4%)
11–30	13 (15.1%)
> 30	35 (40.7%)
Patient population seen (may select more than one)	
Fetal/pregnancy care	30 (34.9%)
Pediatric (age 0–18)	38 (44.2%)
Young adult (age 19–25)	55 (64.0%)
Adult (age > 25)	58 (67.4%)
Practice in a TSC clinic (select one)	
Yes	53 (61.6%)
No	33 (38.4%)
Years in practice (select one)	
0–5	12 (14.0%)
6–10	20 (20.3%)
11–15	12 (14.0%)
16–20	14 (16.3%)
21 +	28 (32.6%)
Gender identify (select one)	
Male	40 (46.5%)
Female	43 (50.0%)
Nonbinary	1 (1.20%)
Prefer not to answer	2 (2.30%)
Race/ethnicity (may select more than one)	
Asian	18 (20.9%)
Black or African American	1 (1.20%)
Hispanic or Latino	2 (2.30%)
White	59 (68.6%)
Prefer not to answer	6 (7.00%)
Other	2 (2.30%)
Primary country of practice (select one)	
United States	71 (82.6%)
Other	15 (17.4%)

### Recognition of risk

The majority of providers (n = 70, 83.3% [CI 73.3–90.3%]) believe that pregnant patients with TSC are at increased risk for maternal health complications. Nearly two-thirds (n = 50, 63.3% [CI 51.6–73.6%]) of providers consider a patient with TSC to have a “high-risk” pregnancy even when there are no clinically significant TSC manifestations.

**Fig. 1:**
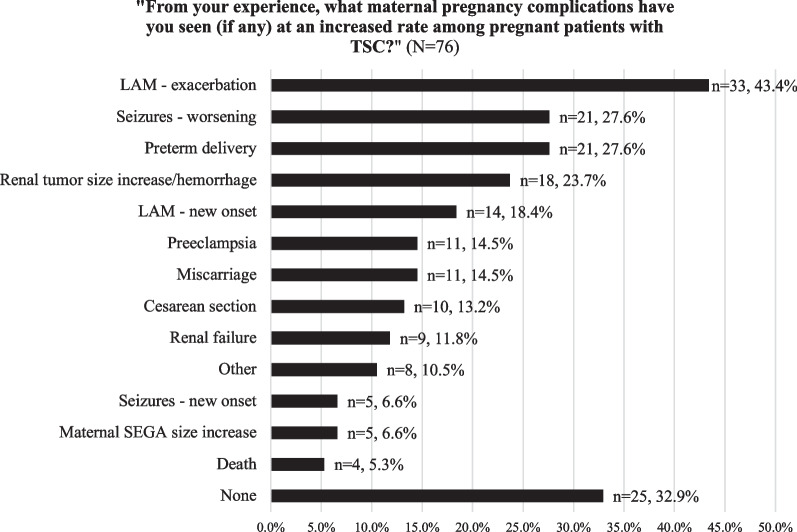
Provider response frequency distribution of provider experience with maternal pregnancy complications (N = 76)

Most providers (n = 70, 88.6% [CI 79.0–94.3%]) believe additional maternal medical management is needed above standard pregnancy care in patients with TSC. This was largely driven by provider experiences with various maternal pregnancy complications at an increased rate in patients with TSC. Fifty-one providers (67.1%) observing at least one complication at an increased rate and at least 1 in 5 providers reporting LAM exacerbation, seizure worsening, preterm delivery, and/or renal AML increase/hemorrhage (Fig. 1). To determine the LAM risks associated with pregnancy, pulmonologists (n = 29) used two major factors to assess disease extent: abnormal pulmonary function tests (PFTs) (n = 25, 86.2%) and/or supplemental oxygen use (n = 23, 79.3%).

### Screenings/evaluations recommended

**Fig. 2:**
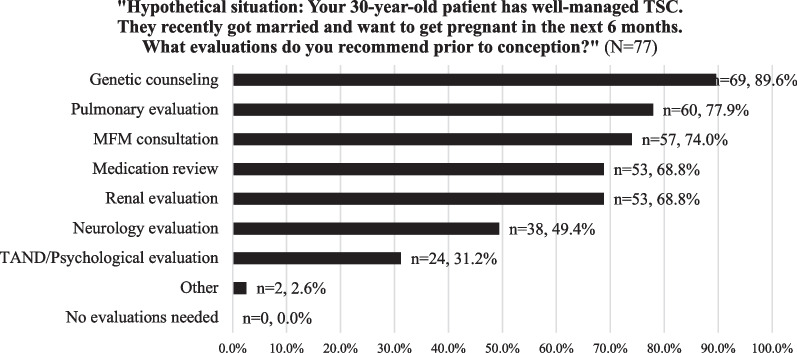
Provider response frequency distribution of provider recommendations for pre-pregnancy evaluations (N = 77)

For well-managed patients with TSC who hope to become pregnant in the next 6 months, the majority of providers recommended genetic counseling, pulmonary evaluation, MFM consultation, medication review, and renal evaluation (Fig. 2). More specifically, 89.7% (n = 26; [CI 78.6–100%]) of pulmonologists recommend obtaining PFTs, 72.4% (n = 21; [CI 56.1–88.7%]) recommend a high-resolution computerized tomography (CT), and 27.6% (n = 8; [CI 11.3–43.9%]) recommend serum vascular endothelial growth factor-D (VEGF-D) quantification to evaluate for the presence of and assess the severity of LAM prior to pregnancy. To monitor LAM progression during pregnancy, 86.2% (n = 25; [CI 73.6–98.8%]) of pulmonologists monitor clinically (history and physical exam) and 72.4% (n = 21; [CI 56.1–88.7%]) perform serial PFTs.

**Fig. 3:**
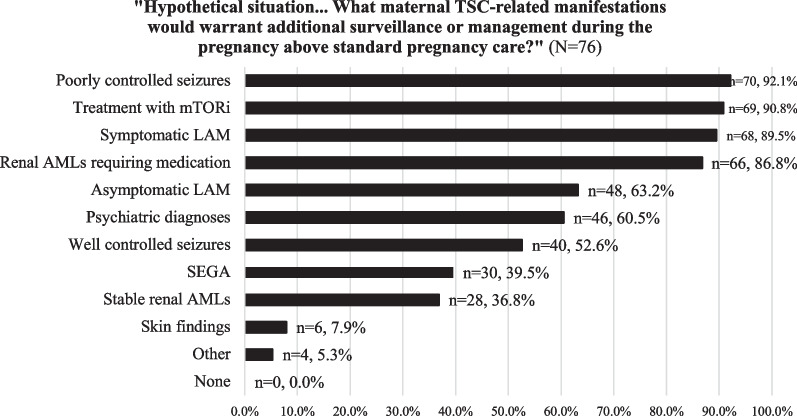
Provider response frequency distribution depicting manifestations that warrant additional surveillance or management during pregnancy (N = 76)

Providers felt that additional care during pregnancy was needed when a patient with TSC had poorly controlled seizures, required treatment with mTORi, had symptomatic LAM, or renal AMLs requiring medication management (Fig. 3). Providers reported performing frequent assessment of medication levels during pregnancy, with monthly laboratory checks being the most commonly utilized frequency (n = 25, 33.8% [CI 23.4–45.8%]), followed by every three months (n = 14, 18.9% [CI 11.1–30.0%]).

**Fig. 4:**
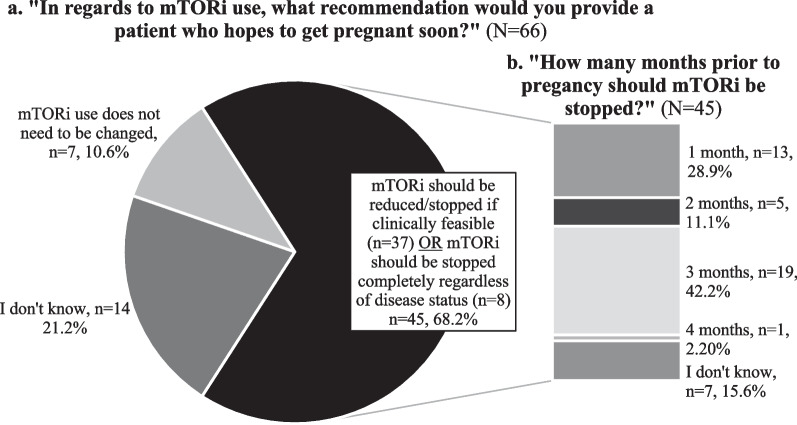
**a** Provider response frequency distribution of recommendation for mTORi use before pregnancy (N = 66) and **b** Provider recommendations of timeframe for reducing/stopping mTORi (N = 45).

**Fig. 5:**
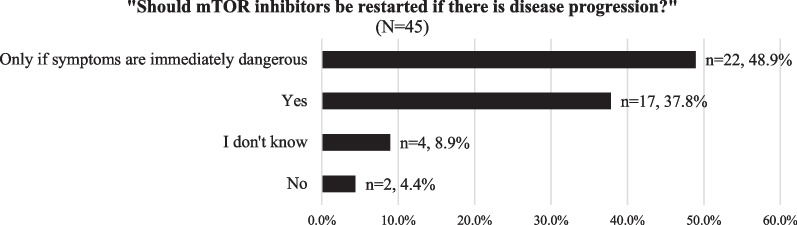
Provider response frequency distribution of recommendation on restarting mTORi given disease progression during pregnancy (N = 45)

Providers were asked about their recommendations regarding mTORi use in patients contemplating pregnancy. Of those providers who reported managing mTORi (N = 66), the majority (n = 45, 68.2%) recommended reducing or stopping mTORi before pregnancy (Fig. 4, a). These 45 providers were asked two follow-up questions: timeframe of stopping mTORi before pregnancy and restarting mTORi if there is disease progression during pregnancy. There was limited consensus on the timeframe to stop mTORi use before pregnancy (Fig. 4b) and the threshold for restarting mTORi in light of disease progression during pregnancy (Fig. 5). However, over 85% of providers suggested resuming mTORi in the event of disease progression during pregnancy (Fig. 5).

In addition, pulmonologists were asked a specific question regarding starting mTORi in patients with TSC-LAM at the time of pregnancy to prevent potential disease progression. The majority of pulmonologists (n = 19, 65.5%) preferred to wait in this scenario, while 5 (17.2%) preferred to proactively start treatment with mTORi.

### Provider/clinic protocols

Most providers (n = 53, 71.6% [CI 59.8–81.2%]) do not have established provider/clinic protocols to manage pregnancy care for patients with TSC. Eleven (14.5%) providers stated that they do have established provider/clinic protocols. To inform their protocols, providers utilized a variety of sources including consultation with other professionals (90.9%, n = 10), professional/clinic experience (81.8%, n = 9), published TSC consensus guidelines (72.7%, n = 8), and current literature/peer reviewed journals (63.6%, n = 7).

## Discussion

Pregnancy complications and prenatal healthcare in patients with TSC is an understudied field. Most of the work in this area, to date, has focused on the effects of TSC on the fetus. However, increased risk of disease progression or new disease-related manifestations have also been described in pregnant patients with TSC [6, 21, 22]. Additional research is needed to clarify complication rates during pregnancy in patients with TSC so that recommendations can be made on appropriate additional screenings.

Results from our provider survey show that TSC providers agree that patients with TSC are at increased risk during pregnancy, but there are several areas where there is no consensus on the level of risk and appropriate mangement. Interestingly, the perception of maternal risk and provider recommendations did not significantly differ with provider specialty or experience.

With only two mTORi on the market, our study did not ask respondents which particular drug they have prescribed to patients of child-bearing potential. The majority of providers would stop mTORi use prior to a planned pregnancy. However, providers were split on the timing of stopping mTORi and what level of disease progression would warrant restarting mTORi. Additionally, there is a wide variance in the work-up conducted prior to and during pregnancy. Altogether these results suggest the need for further study in these areas in order to reach consensus around best practices.

Genetic counseling, which includes providing information to patients and their families regarding the hereditary nature of TSC and understanding recurrence risks, was the most frequently recommended evaluation (n = 69, 89.6%) for patients interested in becoming pregnant. This supports the importance of genetic counseling, as well as the inclusion of genetics as a required specialty for clinics seeking to be designated as a TSC Clinic or TSC Center of Excellence by the TSC Alliance [27]. Similarly, recognition of frequent pulmonary and renal complications in pregnant patients with TSC further supports the need for close collaboration and integration of these specialists in TSC clinics. Our results support the addition of MFM as a core speciality for TSC Clinics and TSC Centers of Excellence.

Our study has demonstrated the uncertainty surrounding the use of mTORi during pregnancy, considering both the drugs’ positive treatment effect on TSC manifestations as well as its unknown human fetal teratogenicity as an FDA category C drug [2, 16]. Given that two-thirds of providers surveyed have observed maternal health complications that generally respond well to mTORi treatment (e.g., LAM, seizures, and renal AMLs), there is a critical unmet need to better understand the safety and efficacy of mTORi use during pregnancy and address the use of such agents in clinical guidelines for both TSC and LAM [28]. Future work will need to investigate the risk of not only using mTORi, but provide data on the risk/benefits of stopping and starting the drug in pregnancy.

It is not surprising that 71.6% (n = 53) of providers do not have established provider/clinic protocols to guide pregnancy care and address potential health complications for patients with TSC given the relative paucity of literature in this area. The lack of established protocols probably means patients are receiving heterogenous care. The current TSC consensus guidelines are limited in their scope when it comes to pregnancy management, as the pregnancy-related recommendations focus on counseling patients on the risk of accelerated lung function decline during pregnancy in patients with LAM [5]. Our study highlights the critical need to draft consensus-based recommendations to guide healthcare providers in optimal management of pregnant patients with TSC. While recognizing that more research is needed to make fully informed recommendations, we propose the following elements be taken into consideration while managing pregnant patients with TSC (Table 2).

**Table 2:** Considerations for the creation of consensus-based guidelines regarding pregnancy management in patients with TSC

1. All pregnancies in patients with TSC should be considered “high-risk” and patients should be counseled on the increased risk of both new onset and worsening of TSC manifestations as well as pregnancy specific complications
2. Preconception genetic counseling and MFM consultation should be provided
3. Medical evaluations for TSC specific manifestations should be done prior to and/or during pregnancy:
i.Pulmonary evaluation (PFT and/or CT)
ii. Renal evaluation (MRI)
iii. Neurologic evaluation, especially as related to seizure control
iv. TAND/Psychological evaluation
4. Providers should provide careful consideration and review of medications prior to and during pregnancy. These decisions, especially concerning mTORi use, should be made via employing shared decision making with the patient and should be individualized to each patient after taking into account the indication(s) for the use of mTORi, underlying disease severity, consequences of treatment interruption, and level of concern regarding potential teratogenic effect to the fetus
i. Circumstances informing the need to stop mTORis and the recommended medication free interval prior to pregnancy
ii. Circumstances that would warrant either maintenance or reinstatement of mTORi use during pregnancy
iii. Seizure medication adjustment during pregnancy including recommendations for the interval of medication blood level checks

There are several limitations and strengths of our analysis. Although this study received a robust response rate and included providers across 10 specialties, the speciality response rate was not analyzed during the survey collection time. Thus, the majority of the providers were either pulmonologists (n = 34, 39.5%) or neurologists (n = 28, 32.6%). Knowing MFM and OBGYN would be a weakness in the target population, a concerted effort to increase the response rate was done (by direct conversations with providers and through asking partnering MFMs to ask colleagues). Yet, these specialities represent a minority (n = 8, 9.30%) of our respondents. Similarly, nephrologists constituted a very small proportion (n = 2, 2.30%) of our respondents, although nephrology being a core TSC clinic group and several providers recognized the risk of renal AML size increase and hemorrhage during pregnancy. While efforts were made to increase provider response rate for MFM and OBGYN providers during the survey time, no active work for nephrologists was made outside of contact at TSC and LAM meetings or through patient advocacy groups.

Given the nature of our study, we recognize that recall bias may have guided provider’s responses/recommendations as negative patient outcomes can overshadow positive outcomes. In addition, providers without a clear understanding of the rate of pregnancy related complications in the general population may have trouble estimating if these complications occur at a higher rate among patients with TSC. The lack of response from MFM and OBGYN providers highlights that the main recommendations for TSC and LAM pregnancy-related care come from groups without these direct expertise. As such, our results are subject to the recall bias of the primary specialities for these patients (pulmonologists and neurologists). Future work to address pregnancy-related risks will need active engagement with MFM and OBGYN to address the bias of other specialty recall of “typical” pregnancy risks. Given that during pregnancy, MFM and OBGYN offices represent the primary point of care for patients, we are hopeful that future research will address this group specifically and highlights the need to include MFM/OBGYN providers into TSC clinical care discussions.

The major strengths of our analysis lie in the diversity of our respondents in various facets such as specialities, experience, country of practice, etc., the clear consensus regarding the consideration of pregnancy in TSC as a high-risk scenario, and the recognition of several specific maternal risks during pregnancy. To our knowledge, our analysis is the first attempt to gather a survey-based assessment from healthcare providers to better understand pregnancy related risks in patients with TSC, report the current practice patterns regarding the surveillance and management of pregnancy in patients with TSC, and highlight the need for consensus-based management guidelines as well as the gaps that need to be filled by future studies.

## Conclusion

The inconsistencies between the perceived need for specialized care during pregnancy, paucity of data in the medical literature, and seemingly heterogenous care being provided to TSC patients before and during pregnancy underscore the urgent need to better understand pregnancy related risks and develop consensus-based guidelines to improve clinical management of this high-risk population.

### Supplementary Information

**Additional file 1.** TSC Questionnaire.

## Data Availability

Anonymized data may be shared by request from qualified investigators. Study data were collected and managed using REDCap electronic data capture tools hosted at the Center for Clinical and Translational Science and Training (CCTST) at the University of Cincinnati and Cincinnati Children’s Hospital Medical Center [ 29, 30 ].

## Abbreviations

TSC: Tuberous sclerosis complex
LAM: Lymphangioleiomyomatosis
mTOR: Mechanistic target of rapamycin
mTORi: MTOR inhibitors
FDA: United States Food and Drug Administration
SEGA: Subependymal giant cell astrocytoma
AML: Angiomyolipoma
MRI: Magnetic resonance imaging
REDCap: Research electronic data capture
QR: Quick response
MFM: Maternal fetal medicine
CI: Confidence interval
OB/GYN: Obstetrics and gynecology
PFT: Pulmonary function test
CT: Computerized tomography
VEGF-D: Vascular endothelial growth factor-D
CCTST: Center for clinical and translational science and training
NIH: National institute of health
CTSA: Clinical and translational science award
