# Supplementary material for: Healthcare provider recognition of pregnancy related risks and management considerations in patients with tuberous sclerosis complex
Source: Orphanet J Rare Dis. 2024 Jan 2;19:4. doi: 10.1186/s13023-023-03015-7 (PMC10763101; doi:10.1186/s13023-023-03015-7)
Supplement: Supplementary file 1 — Additional file 1. TSC Questionnaire. [file 13023_2023_3015_MOESM1_ESM.pdf]

TSC Questionnaire

Questionnaire Introduction:The following questionnaire is part of a Masters in Genetic Counseling thesis work at the University of Cincinnati/Cincinnati Children's Hospital Medical Center. This research aims to describe provider knowledge/understanding of maternal Tuberous Sclerosis Complex (TSC) during pregnancy and potential management changes associated with pregnancy. Of note, the research team recognizes that there is a spectrum of gender identities that often differ from sex. The ability to become pregnant and have children is not restricted to cis-gender females. This research focuses on patients with TSC who have the potential to become pregnant and uses the term "maternal" to describe these patients, regardless of their gender identification. Questionnaire Logistics:Completion of this questionnaire will serve as your consent to participate. Participation is voluntary. There are no inherent risks to completion of the questionnaire and the benefit of participation is the contribution to TSC research. It is estimated that this questionnaire will take less that 10 minutes to complete. Once you answer a question, you will not be able to go back to the previous question. Questionnaire Data Security:This questionnaire will require you to input the zip code or country (if outside of the U.S.) that you practice in. All data will be stored in REDCap and/or on a CCHMC owned and password protected drive and will not be seen by others not involved with the research. Each response will be given a unique study identification number. There will be an option to provide a personal email address. Questions/Clarifications:Please contact our study team if there are any questions/clarifications regarding this questionnaire or the research being conducted. Principal Investigator: Jennifer Glass, MS, LCGC (Jennifer.Glass@cchmc.org)Co-Investigator: Meredith Rose, BA (Meredith.Rose@cchmc.org)

Please answer the following demographic questions:

Are you a healthcare provider?

◯ Yes
◯ No

How many patients with TSC do you see each year on average?

◯ Never seen a patient with TSC
◯ 0-1
◯ 2-10
◯ 11-30
◯ >30

What TSC patient population(s) do you provide care for?

☐ Fetal/pregnancy care
☐ Pediatric (age 0-18)
☐ Young adult (age 19-25)
☐ Adult (age >26)
(May select more than one)

What is your specialty area?

☐ Genetics/Genetic Counseling
☐ Maternal Fetal Medicine
☐ OBGYN
☐ Nephrology
☐ Neurology
☐ Nursing
☐ Oncology
☐ Primary Care
☐ Psychology/Psychiatry
☐ Pulmonology
☐ Other ______
(May select more than one)

How many years have you been in practice?

◯ 0-5
◯ 6-10
◯ 11-15
◯ 16-20
◯ 21+

Do you currently work in a TSC Clinic?

◯ Yes
◯ No

What is your race/ethnicity?	☐ American Indian or Alaska Native<br>☐ Asian<br>☐ Black or African American<br>☐ Hispanic or Latino<br>☐ Native Hawaiian or Other Pacific Islander<br>☐ White<br>☐ Prefer not to answer<br>☐ Other ______<br>(May select more than one)
What is your gender identity?	◯ Male<br>◯ Female<br>◯ Non-binary<br>◯ Prefer not to answer<br>◯ Other
Do you practice in the United States?	◯ Yes<br>◯ No
What zip code do you primarily practice in?	______________________________
What country do you primarily practice in?	______________________________

TSC Questionnaire:	
Do you consider patients with TSC to be at an increased risk for maternal health complications when pregnant? (Maternal health complication is defined as any complication that may increase maternal morbidity or mortality).	◯ Yes<br>◯ No<br>◯ I don't know
What do you use to determine if you do or do not consider patients with TSC to be at an increased risk for maternal health complications when pregnant?	☐ Current literature/peer reviewed journals<br>☐ Current guidelines (2021 Updated TSC Guidelines)<br>☐ Clinic/private practice established protocols<br>☐ Professional experience providing care to patients with TSC<br>☐ Consultation with other professionals<br>☐ Other ______<br>(May select more than one)
Do you believe additional maternal medical management, above standard pregnancy care, is needed during a patient's pregnancy if they have TSC?	◯ Yes<br>◯ No<br>◯ I don't know
Hypothetical situation:<br>A 27-year-old patient comes to your clinic at 11-weeks gestation. They have a genetic diagnosis of TSC, but no presenting symptoms. Do you consider this to be a high-risk pregnancy?	◯ Yes<br>◯ No<br>◯ I don't know
Hypothetical situation:<br>Your 30-year-old patient has well-managed TSC. They recently got married and want to get pregnant in the next 6 months. What evaluations do you recommend prior to conception?	☐ Neurology evaluation<br>☐ Renal evaluation<br>☐ Pulmonary evaluation<br>☐ Maternal Fetal Medicine consultation<br>☐ Medication review<br>☐ TAND/Psychological evaluation<br>☐ Genetic counseling<br>☐ A well-managed patient with TSC does not need additional evaluations<br>☐ Other ______<br>(May select more than one)
Hypothetical situation:<br>Your 30-year-old patient is pregnant and had a normal 12-week ultrasound. The OBGYN calls you to discuss if this patient needs any additional medical management above standard pregnancy care due to her TSC diagnosis. What maternal TSC-related manifestations would warrant additional surveillance or management during the pregnancy?	☐ Renal angiomyolipoma(s) requiring medication<br>☐ Stable renal angiomyolipoma<br>☐ SEGA (subependymal giant cell astrocytoma)<br>☐ Asymptomatic LAM (lymphangioleiomyomatosis)<br>☐ Symptomatic LAM (lymphangioleiomyomatosis)<br>☐ Well controlled seizures<br>☐ Poorly controlled seizures<br>☐ Psychiatric diagnoses<br>☐ Skin findings<br>☐ Treatment with mTOR inhibitors<br>☐ Other ______<br>☐ None of the above<br>(May select more than one)
What fetal screening do you regularly offer for patients with TSC who are pregnant with their own biologic child?	☐ Standard 20-week anatomy ultrasound<br>☐ Additional ultrasounds later in pregnancy<br>☐ Fetal echo<br>☐ Fetal MRI<br>☐ Genetic counseling<br>☐ CVS/Amniocentesis<br>☐ Other ______<br>(May select more than one)

From your experience, what maternal pregnancy complications have you seen (if any) at an increased rate among pregnant patients with TSC?	☐ Miscarriage<br>☐ Renal tumor size increase/hemorrhage<br>☐ Maternal SEGA size increase (subependymal giant cell astrocytoma)<br>☐ Preterm delivery<br>☐ Cesarean section<br>☐ Preeclampsia<br>☐ Renal failure<br>☐ LAM (lymphangioleiomyomatosis) - new onset<br>☐ LAM (lymphangioleiomyomatosis) - exacerbation (pulmonary symptoms including pleural effusion, pneumothoraces, or lung surgeries)<br>☐ Seizures - new onset<br>☐ Seizures - worsening<br>☐ Death<br>☐ Other ______<br>☐ None of the above<br>(May select more than one)
From your experience, what fetal pregnancy complications have you seen (if any) at an increased rate among pregnant patients with TSC?	☐ Polyhydramnios<br>☐ Oligohydramnios<br>☐ IUGR (intrauterine growth restriction)<br>☐ Placental abruption<br>☐ Impaired fetal cardiac blood flow<br>☐ Fetal Arrhythmia<br>☐ Fetal SEGA<br>☐ Fetal tubers<br>☐ Fetal renal AML<br>☐ Fetal renal cyst(s)<br>☐ Other ______<br>☐ None of the above<br>(May select more than one)
Many patients with TSC take oral mTOR inhibitors (ex. Everolimus/Afinitor/ Zortress, Temsirolimus/Torisel, Sirolimus/Rapamune) to treat SEGA, renal angiomyolipomas, refractory epilepsy, and LAM manifestations. What recommendation would you provide a patient who hopes to get pregnant soon?	◯ mTOR inhibitor use does not need to be changed prior to pregnancy<br>◯ mTOR inhibitors use should be stopped completely before becoming pregnant regardless of disease status<br>◯ mTOR inhibitor use should be reduced/stropped if clinically feasible before becoming pregnant<br>◯ I do not manage TSC patients mTOR inhibitors<br>◯ I don't know
How many months prior to pregnancy?	◯ 1 month<br>◯ 2 months<br>◯ 3 months<br>◯ 4 months<br>◯ 5 months<br>◯ 6-12 months<br>◯ >12 months<br>◯ I don't know
Should mTOR inhibitors be restarted if there is disease progression?	◯ Yes<br>◯ No<br>◯ Only if symptoms are immediately dangerous<br>◯ I don't know

How often do you check medication/serum (mTOR inhibitor, epilepsy medication, etc.) levels during a TSC patient's pregnancy?	☐ Every month<br>☐ Every 3 months<br>☐ Every 6 months<br>☐ I do not prescribe medications<br>☐ I don't know<br>☐ Other ______<br>(May select more than one)

The following question(s) are based on your specialty area:

When a patient with TSC becomes pregnant, how is the TSC Clinic involved in their prenatal care?

____

Do you give different advice to your patients with sporadic LAM versus TSC-associated LAM regarding risks related to pregnancy?

- ◯ Yes
- ◯ No
- ◯ I don't know

What LAM screening would you recommend prior to pregnancy?

- ☐ VEGF-D
- ☐ High resolution Chest CT
- ☐ Pulmonary Function Test (PFT)
- ☐ Other ______

(May select more than one)

How would you monitor LAM progression during pregnancy?

- ☐ Serial PFTs
- ☐ Clinically (history and physical only)
- ☐ High resolution chest CT
- ☐ Serial VEGF-D
- ☐ Other ______
- ☐ I don't know

(May select more than one)

PFTs every _____

- ◯ 1 month
- ◯ 2 months
- ◯ 3 months
- ◯ 4 months
- ◯ 5 months
- ◯ >5 months
- ◯ I don't know

CTs every _____

- ◯ 1 month
- ◯ 2 months
- ◯ 3 months
- ◯ 4 months
- ◯ 5 months
- ◯ >5 months
- ◯ I don't know

VEGF-D every _____

- ◯ 1 month
- ◯ 2 months
- ◯ 3 months
- ◯ 4 months
- ◯ 5 months
- ◯ >5 months
- ◯ I don't know

Would you start mTOR inhibitor use in a patient with TSC-LAM at the time of pregnancy to prevent accelerated disease progression?

- ◯ Yes
- ◯ No
- ◯ I don't know
- ◯ Other ______

What LAM manifestations would make you discourage a patient from becoming pregnant?

- ☐ History of recurrent pneumothorax
- ☐ Supplemental oxygen requirement
- ☐ Chylothorax
- ☐ Disease extent as suggested by PFTs
- ☐ Disease extent as suggested by chest CT
- ☐ Other ______

(May select more than one)

What threshold of PFTs would make you say pregnancy is unsafe?

FEV1 DLCO Other

If a patient presents to your clinic with TSC or a fetus is suspected to have TSC, do you involve a TSC Clinic in the prenatal care?

- ◯ Yes
- ◯ No
- ◯ Sometimes
- ◯ I don't know

Briefly describe how your practice works with a TSC clinic to care for patients who have TSC or when TSC is suspected in a fetus.

____

Do you refer patients with TSC or a fetus suspected to have TSC to a TSC clinic after birth?

____

In what scenarios, and how, do you involve TSC clinics in the care of patients with TSC or when TSC is suspect in a fetus?

____

Do you or your clinic/practice have established protocols or standard procedure, either written or unwritten, to manage pregnancy care for patients with TSC?

- ◯ Yes
- ◯ No
- ◯ I don't know

What information did your clinic/practice use to establish protocols?

- ☐ Published TSC Consensus Guidelines
- ☐ Current literature/peer reviewed journals
- ☐ Professional/Clinic experience (pregnancy outcomes, maternal complications, etc.)
- ☐ Consultation with other professionals
- ☐ Other ______
- ☐ I don't know

(May select more than one)

Please briefly describe key elements of your protocol/standard procedures.

____

Would you be willing to be contacted to further discuss your protocol/standard procedures?

- ◯ Yes
- ◯ No

What is your email address?

____

Thank you for taking the time to complete this questionnaire

Do you have any other thoughts about this questionnaire or topic that you would like to share? ________________________________
